# Change of spermatophyte family diversity in distribution patterns with climate change in China

**DOI:** 10.1016/j.heliyon.2024.e28519

**Published:** 2024-03-30

**Authors:** Yanzi Wang, Zhenhong Wang

**Affiliations:** a Key Laboratory of Subsurface Hydrology and Ecological Effects in Arid Region, Ministry of Education, Chang'an University, Xi'an, Shaanxi Province, 710054, China; b School of Water and Environment, Chang'an University, Xi'an, Shaanxi Province, 710054, China

**Keywords:** Chinese nature reserves, Global climate change, Scenario analysis, Spatial simulation model, Spermatophyte family diversity

## Abstract

The global climate is undergoing extraordinary changes, profoundly influencing a variety of ecological processes. Understanding the distribution patterns and predicting the future of plant diversity is crucial for biodiversity conservation in the context of climate change. However, current studies on predictive geographic patterns of plant diversity often fail to separate the effects of global climate change from other influencing factors. In this study, we developed a spatial simulation model of spermatophyte family diversity (SSMSFD) based on data collected from 200 nature reserves covering approximately 1,500,000 km^2^, where direct anthropogenic disturbances to plant diversity and the surrounding environment are absent. We predicted the spermatophyte family diversity for all provinces in China in 2020, 2040, and 2080, considering the impacts of global climate change. On average, China currently exhibits 118 plant families per 25 km^2^, with a decreasing trend from southeast to northwest. When considering only the effects of global climate change, excluding direct anthropogenic disturbances, our results indicate that under the Shared Socioeconomic Path Scenarios (SSPs) 245 and 585, spermatophyte family diversity is projected to slowly increase in most Chinese provinces from 2021 to 2080. Notably, the increase is more pronounced under SSPs585 compared to SSPs245. Global climate change has a positive effect on plant diversity, in contrast to the negative impact of anthropogenic disturbances that often lead to declines in plant diversity. This research highlights the contrasting outcomes of future plant diversity under the sole influence of global climate change and the significant negative effects of anthropogenic disturbances on diversity.

## 1 Introduction

Vegetation, one of the most crucial elements of terrestrial landscapes, serves as the foundation of terrestrial ecosystems [1]. The distribution pattern of spermatophyte species diversity, which provides essential sites and energy for the growth and reproduction of other biological species, is of great importance [2]. This distribution pattern, along with biodiversity and ecosystem function, can be directly influenced by climate and its regional and temporal changes [3]. Factors such as temperature, precipitation, latitude, longitude, elevation, and area play a significant role in shaping the distribution of tree species [4]. Precipitation and temperature, in particular, have been identified as primary determinants of plant diversity and distribution [[5], [6], [7]]. With the effects of global climate change, it is predicted that the average temperature will increase, causing shifts in the suitable distribution areas of plant species towards higher altitudes [8]. In addition to climate factors, land use/cover change, topography, interspecific interactions, and human activities also have varying degrees of impact on plant diversity. Land use/cover change, driven by global change, is a major driver that directly affects Earth's biodiversity [[9], [10], [11]]. Topography, especially in mountainous areas with complex terrain, plays an important role in localized vascular plant diversity patterns and supports high plant biodiversity and local special species [12]. Global warming has led to an increase in surface temperatures, which has influenced vegetation structures and dynamics, including species richness, composition, biomass, and productivity [13].

Simulating the spatial distribution of plant diversity and quantitatively analyzing species diversity and its influencing factors present important and challenging research areas [14]. Over the past few decades, scholars have studied the distribution patterns of many biological species under climate change using various methods. These methods include GIS and remote sensing, generalized additive models (GAM), Maxent, Bioclim, domain models, and mathematical and statistical modeling (neutral theory models, ecological niche models, prioritization models, segmentation line segment models, and ecological niche overlap models). For example, Cetin combined GIS and remote sensing techniques to analyze the bioclimatic factors affecting multiple ecological processes with land use/cover change [15,16]. Shen et al. developed a model for forecasting the distribution of vegetation type diversity using community-habitat factor regression analysis combined with GIS-supported vegetation spatial pattern modeling. Zerger et al. used GAM to map vegetation distribution in two 2600 km^2^ areas in western New South Wales. Zou et al [17]. explored the relationship between species diversity of Rosaceae and environmental variables using generalized linear models based on phytobotanical records. Varol estimated the current and future geographic distribution of European ash and fir in Turkey using the entropy method [18,19]. Xu estimated the potential distribution area of *Chionis alba* using two ecological models, Bioclim and Domain, integrated in Diva-Gis [[20], [21], [22]]. Li utilized mathematical-statistical models to explain forest community structure and fit the species diversity of plant communities at different successional stages, such as in the Huoshan greasy pine forest in Shanxi and Changbai Mountain [23,24].

Currently, most researchers focus on simulating the spatial distribution of plants and animals by gathering data on flora, fauna, and environmental factors to study species diversity, underlying mechanisms, and diversity prediction at various scales, from local to regional and even intercontinental. For instance, Rahbak and Graves investigated the influence of several environmental variables on the gradient of species richness within differently sampled plots using the distribution data of 2869 bird species in South America [25]. Xie revealed the horizontal distribution pattern and statistical mechanism of woody plant species richness at six distinct sizes using 11,405 woody plant species in China [26]. However, there is limited research on predicting diversity patterns outside observed regions using spatial simulation models, where key influencing factors are considered while controlling or keeping other factors unchanged [27,28]. Such studies can help clarify the effects and underlying mechanisms of key factors on diversity. Additionally, most studies have focused on predicting the spatial pattern of individual species or a few species in localized areas, without addressing the prediction of spatial patterns for entire biological groups on a larger scale. Predicting the distribution of biological groups under global climate change can provide a comprehensive understanding of biodiversity changes and has significant implications for biodiversity conservation and nature reserve planning. Moreover, the future prominence of global climate change will significantly impact life on Earth, particularly plants, due to their limited migration mechanisms [29]. Nature reserves, with relatively less anthropogenic interference, preserve native plant diversity and can accurately reflect the spatial distribution patterns of plant diversity under future global climate change. Therefore, predicting future plant diversity using nature reserves is a promising approach.

To address these research gaps, we conducted a large-scale prediction of the spatial distribution patterns of most species within a specific biological group under current and future climate change conditions in China. We collected data on spermatophyte family diversity, climatic and geographical variables, from 200 natural reserves that have been sealed off and under control for one to several decades, ensuring no direct human disturbance. These reserves are located in different areas ranging from 73°33′ E to 135°05′E and 3°51′N to 53°33′N in China. We screened the climatic and geographical variables affecting spermatophyte family diversity patterns, eliminating highly collinear variables, and identified key variables for establishing a Spatial Simulation Model for Spermatophyte Family Diversity (SSMSFD) at a regional scale (Table 1). Using climate change data and the SSMSFD, we predicted the regional differences in spermatophyte family diversity for all provinces in China over the next 80 years. Our study is premised on two assumptions: (1) only changes in climatic and geographical factors and related processes such as species invasion and interactions (since 80 years is insufficient for speciation by evolution) affect diversity patterns in nature reserves, allowing SSMSFD to isolate the effects of climate change on diversity; (2) global climate change will result in a more humid and hotter climate in the north and west of China, and therefore, the diversity of southern and eastern nature reserves can be used to simulate the future diversity of northern and western ones using SSMSFD. This study aims to answer three scientific questions: (1) How can predictive modeling of changes in spatial patterns of plant diversity at large scales be developed? (2) How do the patterns of spermatophyte family diversity change under global climate change? (3) What factors are most likely to cause a decline in spermatophyte family diversity?

**Table 1:** Contribution rate of six influential variables from different groups to spermatophyte family diversity This table presents the contribution rates of six influential variables belonging to different groups towards spermatophyte family diversity. The variables were carefully selected based on their significance in influencing the diversity of spermatophyte families. The table provides valuable insights into the relative importance of each variable in shaping the patterns of spermatophyte diversity.

Environmental variables	Description	Contribution rate/%	Optimal regression model
bio2	Monthly mean diurnal temperature difference	−74.9	Linear
bio6	Coldest monthly minimum temperature	76.7	Linear
bio10	Warmest quarterly mean temperature	44.9	Linear
bio12	Average annual precipitation	80.1	quadratic
bio15	Coefficient of variation of precipitation	72.5	Linear
bio23	Latitude	75.4	quadratic

Note: All variables showed significant correlation with spermatophyte family diversity (P < 0.01).

## 2 Materials and methods

### 2.1 Methodological framework

**Fig. 1:**
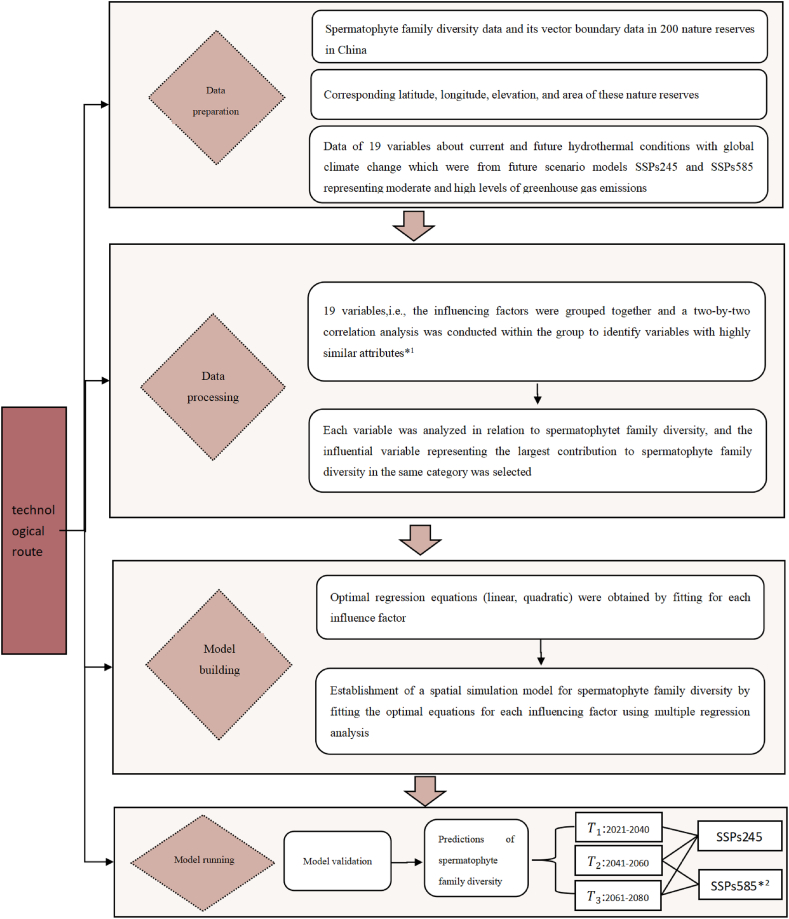
Technology Roadmap. *1: Variables were categorized into groups including temperature, temperature change, precipitation, precipitation change, and geospatial characteristics. Two-by-two correlation analyses were performed within each group to minimize redundancy among similar variables and identify representative variables with the highest contribution to plant diversity. *2: Hydrothermal data for future time periods ($T_{1}$-$T_{3}$) were obtained from the Beijing Climate Center_Climate System Model (BCC_CSM) developed by the National Climate Center of China (NCC). The model utilized the SSPs245 and SSPs585 scenarios, representing pathways with medium and high greenhouse gas (GHG) emission concentrations, respectively. Note: SSPs refers to Shared Socioeconomic Pathways, which are scenarios that describe different potential future socioeconomic conditions and their implications for greenhouse gas emissions and climate change.

The overall methodology of this study, which includes data collection, data processing, model construction, model validation, and model application, is illustrated in Fig. 1. The specific steps are as follows: (1) Data Collection: Spermatophyte family diversity data, along with geographical data (longitude, latitude, elevation, and area) for nature reserves, were sourced from the official websites of the reserves or from relevant literature. Bioclimatic data, comprising of 19 factors, were obtained from the Beijing Climate Center_Climate System Model (BCC_CSM), developed by the National Climate Center of China. Detailed data can be found in the annex. (2) Data Processing: We conducted two-by-two correlation analyses of bioclimatic and geographical factors for 100 nature reserves (selected from a total of 200). After eliminating variables with high redundancy, key representative variables were identified. (3) Model Construction: Linear and quadratic regressions were used to fit family diversity to the key variables. Multiple regression analysis was then employed to construct the final model. (4) Model Validation: Data from the remaining 100 nature reserves, not used in model construction, were utilized to validate the model by calculating the error. (5) Model Application: GIS software was used to partition China into numerous 5 × 5 square kilometer units. The model was then applied to these units, and the results are presented graphically.

### 2.2 Data collection

**Fig. 2:**
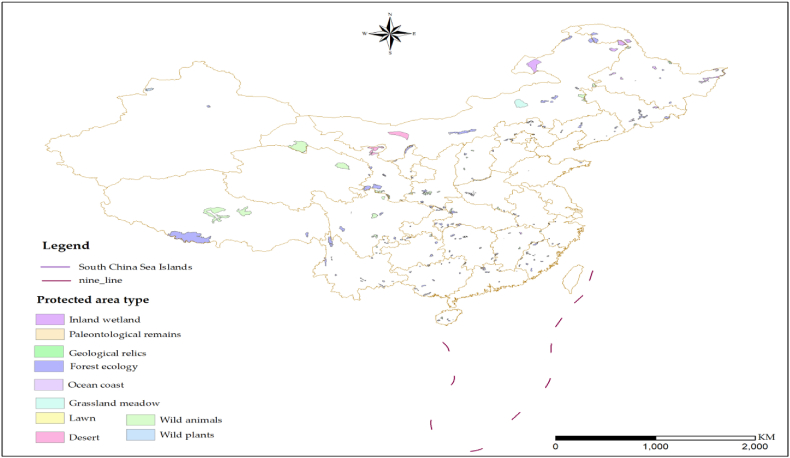
Location of nature reserves for data collection and model validation.

Spermatophyte family diversity data for 200 nature reserves in China were gathered from various literature sources and official nature reserve websites (Fig. 2). Detailed data and sources for each reserve are provided in Supplemental File 1. Area and boundary vector data for the reserves were sourced from the China Nature Reserve Specimen Resource Sharing Platform (http://www.papc.cn). Data for 19 bioclimatic variables, reflecting past, current, and future hydrothermal conditions (T_0: 1981–2000, T_1: 2021–2040, T_2: 2041–2060, T_3: 2061–2080), were obtained from future scenario models SSPs245 and SSPs585, representing moderate and high levels of greenhouse gas emissions. These were obtained from the BCC_CSM developed by the National Climate Center of China (NCC) [30]. The data were extracted using the spatial analysis tool in ArcGIS 10.8 and have a resolution of 30” (about 1 km) for the current and future time periods (T_0-T_3) for the 19 bioclimatic variables in the WGS84 coordinate system. Geospatial data, including elevation (bio20), area (bio21), longitude (bio22), and latitude (bio23), were obtained from the Geospatial Data Cloud of the Chinese Academy of Sciences (http://www.gscloud.cn).

## 3 Data processing

The 23 bioclimatic and geospatial variables were divided into five groups representing temperature, temperature change, precipitation, precipitation change, and geospatial characteristics. Two-by-two correlation analyses within each group were conducted to eliminate redundancy and identify representative variables. All variables within the same group exhibited a correlation coefficient of up to 0.8 following correlation analysis and F-test. Univariate regression analysis was then conducted using the spermatophyte family diversity data and the variables from each group, with the former serving as the dependent variable and the latter as the independent variables. The regression models took the form y = A + Bx for linear models and y = A + Bx^2^ for quadratic models, where y represents the spermatophyte family diversity, x represents the effect variable, and A and B are coefficients. The variable with the highest R^2^ in each group was selected as the influential variable for the final fitting of SSMSFD (Table 1) [31].

This figure illustrates the spatial distribution of nature reserves used for collecting data on spermatophyte family diversity, as well as for establishing and validating the model. These nature reserves cover a total area of 1,471,400 km^2^, which accounts for approximately 14.08% of China's total land area. The nature reserves were strategically selected to represent different regions and ecosystems across the country, ensuring a comprehensive coverage of the study area. These reserves provide valuable data that have been utilized to develop and validate the SSMSFD model, enabling the assessment and prediction of spermatophyte family diversity patterns at a regional scale.

### 3.1 SSMSFD

Based on all the equations of regression to each influential variable obtained above, and the data of 100 nature reserves, multiple nonlinear regression (including linear and quadratic equations) was performed using SPSS software to finally obtain SSMSFD, i.e., Equation (I).

(1) $$\text{SF}=\text{Cons}+\alpha \text{bio}2+\beta \text{bio}6+\gamma \text{bio}10+\left(\delta_{1}\text{bio}12+\delta_{2}\text{bio}12^{2}\right)+\varepsilon \text{bio}15+\left(\epsilon_{1}\text{bio}23+\epsilon_{2}{\text{bio}23}^{2}\right)$$

where SF is spermatophyte family diversity (s); Cons is a constant; $\alpha ,\beta ,\gamma ,\delta_{1},\delta_{2},\varepsilon ,\epsilon_{1}\;\text{and}\;\epsilon_{2}$ are the coefficient of each influencing variable. The cons = 174.621, α = -0.189, β = 0.437, γ = -0.116, $\delta_{1}$ = 0.089, $\delta_{2}$ = -2.611 $\times 10^{-5}$, ε = -0.312, $\epsilon_{1}$ = -3.428, $\epsilon_{2}$ = 0.034. The R2 of Equation (1) is 0.842, and which is at a confidence level of 99%. This equation served as the foundation for simulating and analyzing the current and future spatial distribution of spermatophyte family diversity in each Chinese province.

### 3.2 Model verification

The data of the influential variables collected from other 100 nature reserves were used to predict the spermatophyte diversity based on Equation (1), and the predicted values were compared with the actual diversity by the validation formula (2).

(2) $$S_{\text{Error}}=\frac{1}{n}\sum_{n}^{i=1}\left[\left|\text{Valu}e_{\text{is}}-\text{Valu}e_{\text{it}}\right|\right]$$

where $S_{\text{Error}}$ is the simulation error of spermatophyte family diversity with Equation (1); n is the number of nature reserves; $\text{valu}e_{\text{im}}$ is the simulated value of spermatophyte family diversity in the i-th nature reserve; $\text{valu}e_{\text{ia}}$ is the actual value of spermatophyte family diversity in the i-th nature reserve.

According to the findings of error analysis, the overall simulation error of spermatophyte family diversity for 100 nature reserves is 16.6% compared with the actual value (Supplemental file 2). This demonstrated that the simulation outcomes of SSMSFD were essentially consistent with data in the verification area, which could be applied to the prediction of the spatial pattern of spermatophyte family diversity in other regions of China.

### 3.3 Scenario design and prediction method

Each province in China was gridded on a standard 5 km × 5 km grid cell. Beijing, Tianjin, Shanghai, Chongqing, Macao, and Hong Kong had been incorporated into Hebei, Jiangsu, Sichuan and Guangdong provinces, respectively, because of their relatively small size and serious urbanization, while Taiwan, about 30 thousand km^2^, has not been predicted because of no basic data of nature reserves. The latitude and longitude of the center point of each grid were determined by ArcGIS. The values of all influential variables within each grid cell were extracted using ArcGIS from scenarios SSPs 245 and SSPs 585 ranging from $T_{1}\text{to}\;T_{3}$ [32]. Then the values of the influential variables for each cell were applied to Equation (1) to predict the diversity of the cell.

## 4 Result

### 4.1 Spatial distribution pattern of spermatophyte family diversity

**Fig. 3:**
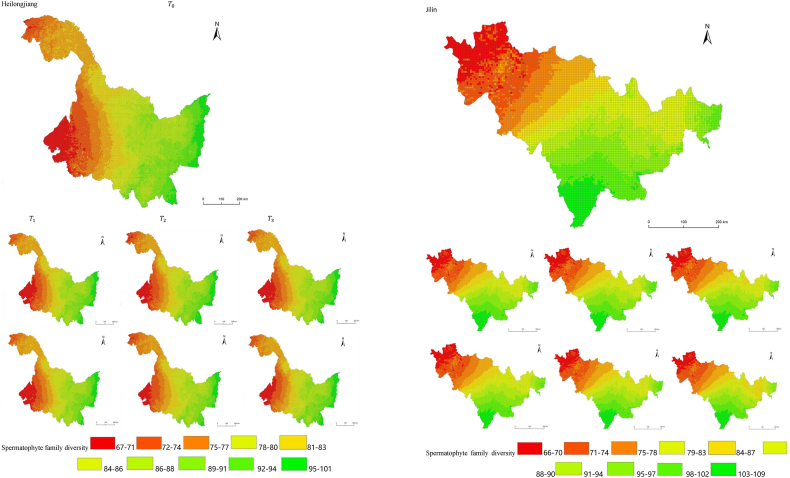

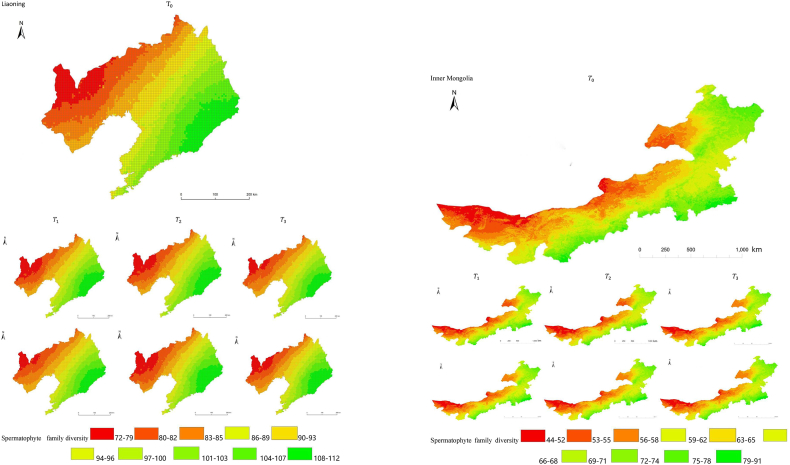

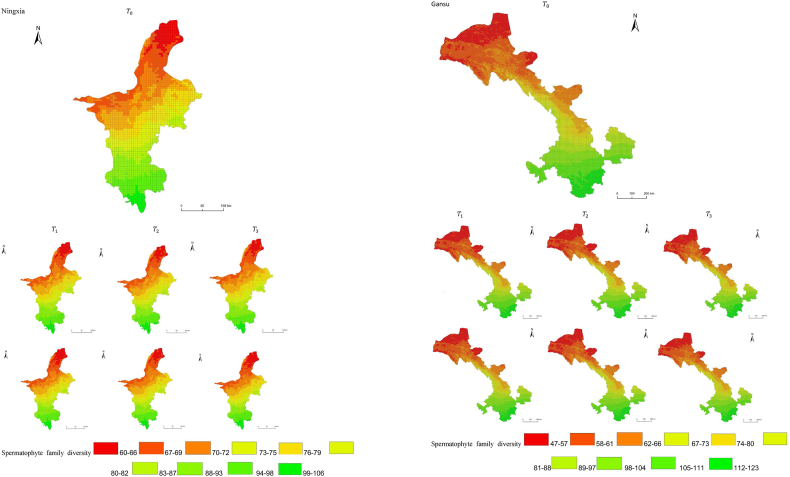

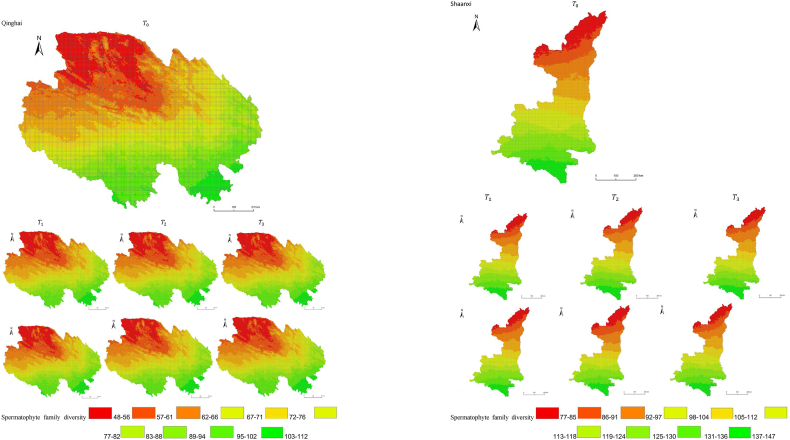

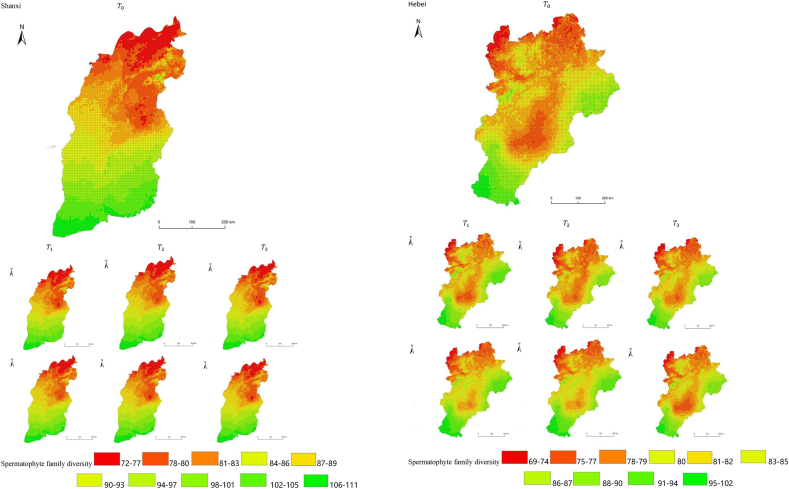

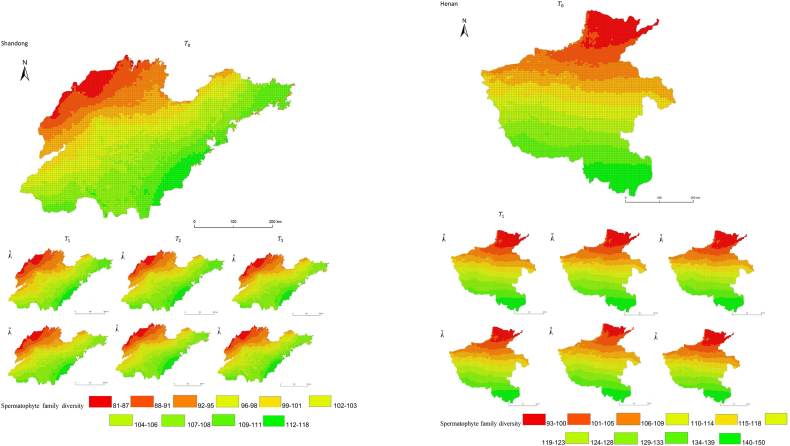

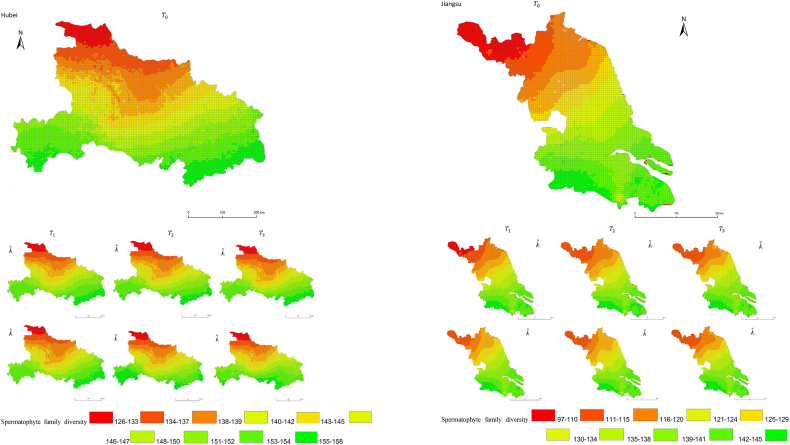

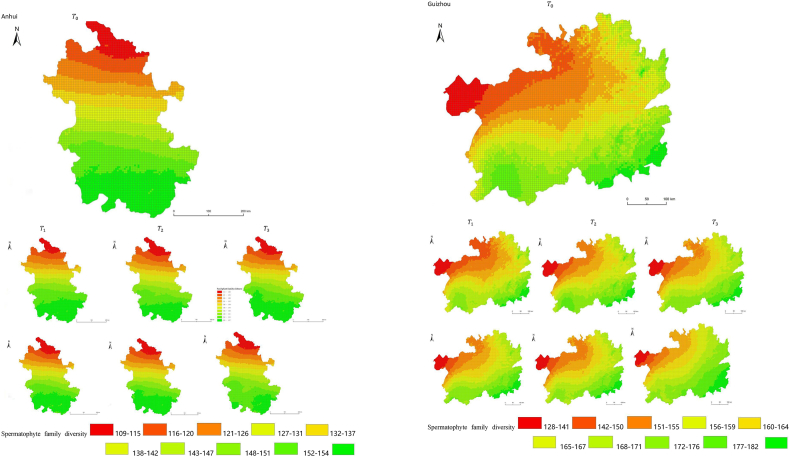

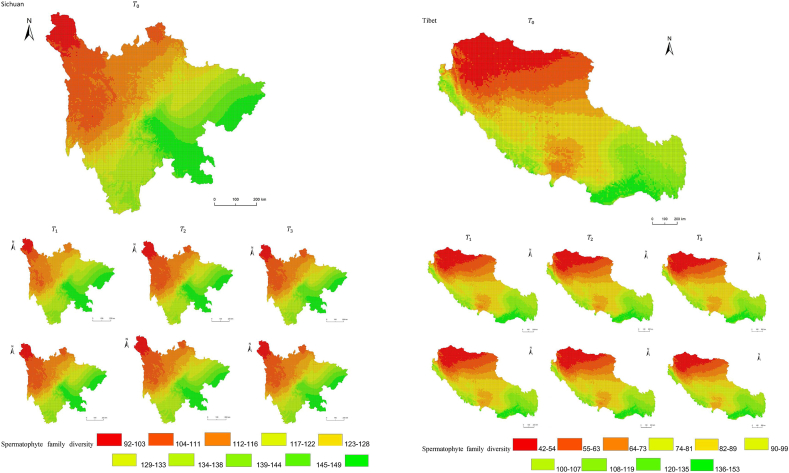

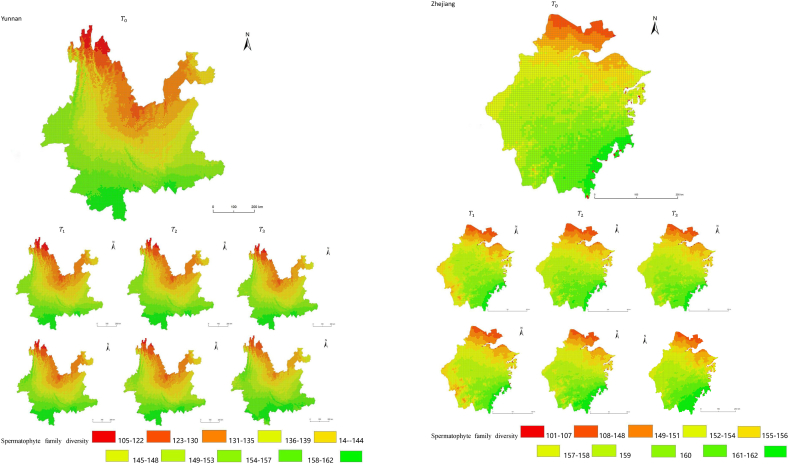

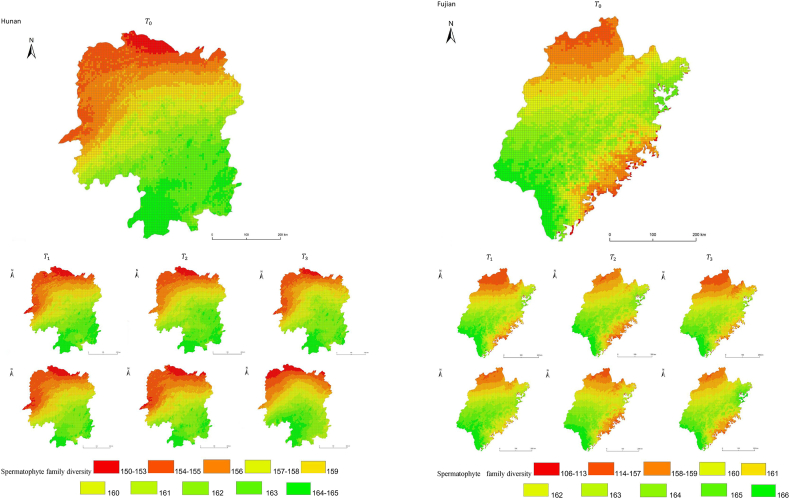

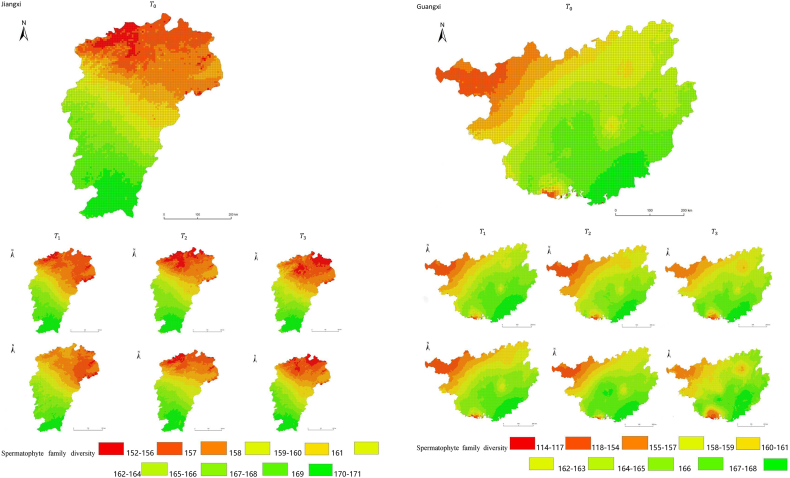

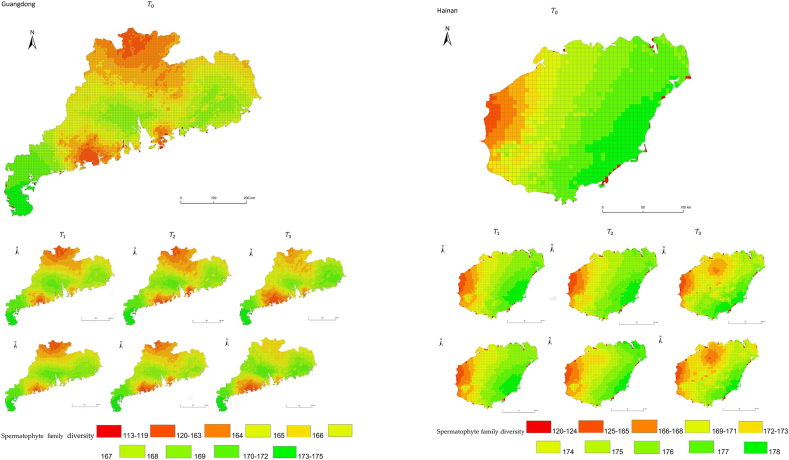

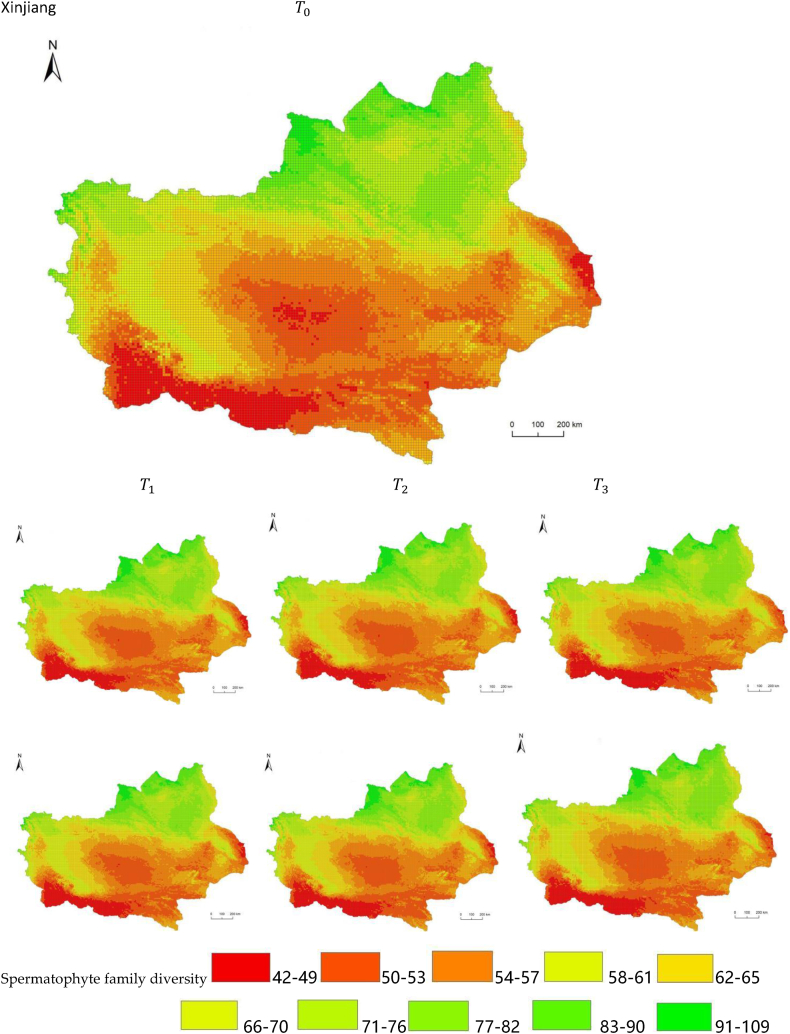
Spatial distribution of spermatophyte family diversity from T_1 to T_3 under SSPs245 and SSPs585 scenarios in China. The map shows the diversity of spermatophyte families using different colors, with specific values provided in the legend. The transition from red to green indicates a gradual increase in diversity. A total of 27 provinces were included in the predictions, with Xinjiang being the largest province, covering a total area of 1.6649 million km^2^.

The simulation results revealed a wide spatial variation in the diversity of spermatophyte families across each province of China. The northern regions of Inner Mongolia and Xinjiang exhibited the lowest average diversity, with 64.54 families of plants per 25 km^2^, while the southern regions of Sichuan and Yunnan showed the highest average diversity (Fig. 3). Interestingly, the average diversity of Ningxia was similar to Gansu in the same climatic zone, despite the vast difference in their respective areas (66,400 km^2^ and 425,800 km^2^), suggesting that area did not significantly influence average diversity (P > 0.05) compared to climatic variables. In terms of spatial distribution, the number of plant families gradually decreased from southeast to northwest, encompassing provinces such as Heilongjiang, Jilin, Liaoning, Inner Mongolia, Gansu, Qinghai, Shandong, Jiangsu, Guizhou, Sichuan, Tibet, Guangxi, and Hainan (Fig. 3). In the following provinces: Ningxia, Shaanxi, Shanxi, Hebei, Henan, Hubei, Hunan, Anhui, Yunnan, Zhejiang, Fujian, Jiangxi, and Guangdong, the number of plant families exhibited a gradual decrease from south to north. The decrease in the number of families followed a clear zonal pattern in terms of latitude and longitude.

### 4.2 Scenario analysis of spermatophyte family diversity

**Fig. 4:**
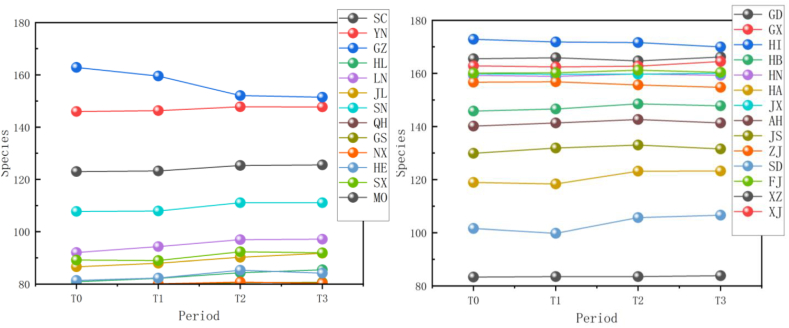
Changes of spermatophyte family diversity in different provinces from $T_{1\;}\mathrm{to}\;T_{3}$**under the SSPs245 scenario.** SC: Sichuan; YN:Yunnan; GZ:Guizhou; HL:Heilongjiang; LN:Liaoning; JL:Jilin; SN:Shaanxi; QH:Qinghai; GS:Gansu; NX:Ningxia; HE: Hebei; SX: Shanxi; MO: Inner Mongoria; GD:Guangdong GX:Guangxi HI:Hainan; HB:Hubei; HN:Hunan; HA:Henan; JX:Jiangxi; AH:Anhui; JS:Jiangsu; ZJ:Zhejiang; SD:Shandong; FJ:Fujian; XZ:Xizang; XJ:Xinjiang.

**Fig. 5:**
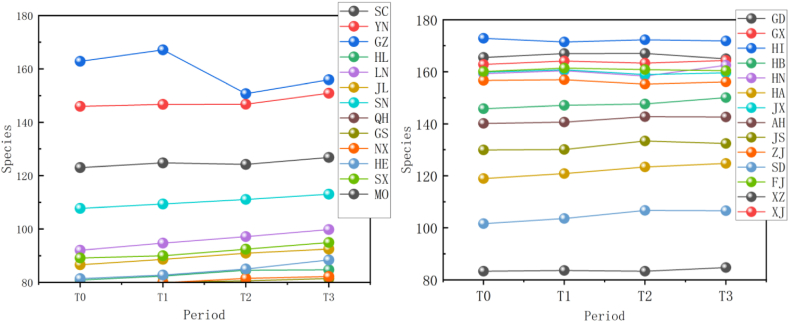
Changes of spermatophyte family diversity in different provinces of China from $T_{1\;}\mathrm{to}\;T_{3}$ under the SSPs585 scenario.

The simulation findings under the two scenarios, SSPs245 and SSPs585, showed an overall increasing trend in spermatophyte family diversity over time in most provinces of China (Fig. 4, Fig. 5, Supplemental file 3 and 4). In Shandong, the diversity of spermatophyte families exhibited a significant change, with an average increase of 2.01%, 5.91%, and 0.85% per 25 km^2^ from T_0 to T_1, T_1 to T_2, and T_2 to T_3 under the SSPs245 scenario. However, Guizhou, Hainan, and Zhejiang experienced a slight decreasing trend in spermatophyte family diversity, with Guizhou exhibiting a more significant decrease of 2.01%, 4.38%, and 0.41% per 25 km^2^ during the three periods under the SSPs245 scenario. Under the SSPs585 scenario, Guizhou displayed a fluctuation of increase, decrease, and increase again, with an average increase of 2.61%, −9.49%, and 3.49% per 25 km^2^ during the three periods, respectively. Notably, spermatophyte family diversity in all provinces showed greater changes under the SSPs585 scenario compared to the SSPs245 scenario.

## 5 Discussion

### 5.1 Spatial simulation of spermatophyte diversity pattern based on the data of natural reserves

Global climate change is an extraordinary event that affects the structure and function of ecosystems worldwide. Spermatophyte family diversity plays a crucial role in ecosystem structure. While numerous studies have explored the effects of climate change on ecosystem structure, including patterns of species diversity, species invasion, and functional group diversity, limited research has focused on the regional changes in biological group diversity, such as spermatophyte family diversity. To address this gap, we combined spermatophyte family data from a large number of nature reserves with future bioclimate scenarios to develop the SSMSFD model and predict the distribution pattern of spermatophyte family diversity. The predicted outcomes represent the potential diversity of spermatophytes in ecosystems or landscapes under conditions excluding direct human disturbance but considering climate change. These ecosystems and landscapes may be nature reserves of forests, grasslands and wetlands, or may be the ecosystems and landscapes that resemble the nature reserves or transit from farmland to natural ecosystems due to strictly ecological redline management in China. The model developed in this study can be used for quantitative analysis of regional plant diversity and its changes in response to global climate change, as well as for analyzing the underlying mechanisms of plant diversity. However, there is still room for improvement in the SSMSFD model to reduce the current predictive errors (16.6%).

### 5.2 Spatial change of spermatophyte family diversity across different periods in China

The diversity of spermatophyte families exhibited a declining trend from south to north and from southeast to northwest in China, consistent with the widely acknowledged "diversity pattern of species diversity decreasing from the equator with increasing latitude" or horizontal zoning [[33], [34], [35]]. According to the climate stability hypothesis and Rapoport's law, as latitude increases, the temperature difference from south to north tends to increase, while species richness decreases. This study further validates this hypothesis through quantitative simulation of spermatophyte family diversity patterns across different provinces in China [36,37]. The overall spermatophyte family diversity in China is projected to increase based on the simulation of spermatophyte diversity from T_1 to T_3 under two future climate scenarios, SSPs245 and SSPs585. Specifically, under the SSPs585 scenario, a larger increase in spermatophyte family diversity is expected compared to the SSPs245 scenario. This finding contradicts general knowledge and may be met with skepticism from other ecologists. However, it is important to note that the results only indicate an increase in spermatophyte family diversity under a single climatic change in the future, assuming the exclusion of anthropogenic disturbances such as land-use change, deforestation, and severe pollution leading to mortality. This assumption holds true for all nature reserves and ecological restoration regions that have been protected by ecological redline management, covering approximately 60% of China's area. It is widely recognized that warm and humid regions can support high plant diversity, while anthropogenic disturbances can severely impair plant diversity. The predictive results of SSMSFD, which indicate an increase in plant diversity with global climate change after excluding anthropogenic disturbances, are highly probable and can provide valuable references for ecosystem management in China and globally.

### 5.3 Key factors influencing spermatophyte family diversity

Correlation and regression analyses conducted during the modeling process revealed that average annual precipitation had the most significant effect on spermatophyte family diversity, explaining 80.1% of the variation. Among all the selected influencing factors in the model, only the monthly mean diurnal temperature difference showed a negative effect, indicating that greater temperature differences within a month were associated with lower diversity of seed plant families. These climatic factors vary over time, indicating climate change as a driving force behind the increase in regional spermatophyte family diversity as predicted by SSMSFD. The SSMSFD model has been validated using actual data and can accurately predict the diversity of spermatophyte families under various future climate change scenarios on a macro scale. It is important to note that most studies on the spatial distribution of species richness have reported a decrease in species richness when considering the combined effects of climate change and anthropogenic disturbance. In this study, we specifically excluded the effects of anthropogenic disturbance by selecting nature reserves that are not directly influenced by human activities and focused solely on the effects of climate change. Therefore, the present study assumes that the effects of anthropogenic disturbances on spermatophyte family diversity are excluded, while only the effects of future climatic conditions are considered. Anthropogenic disturbances such as land use/cover changes, deforestation, grazing, and pollution exert significant pressure on plant habitats and the plants themselves, often leading to species extinction as individual plants struggle to adapt to environmental changes. Thus, different forms of anthropogenic disturbance contribute to a decrease in spermatophyte family diversity compared to climate change alone. Under conditions without human disturbance, climate change, primarily driven by changes in bioclimatic factors, leads to an increase in plant diversity. In natural ecosystem management, it is crucial to focus on controlling anthropogenic disturbances in various forms to minimize their impact. However, it is important to acknowledge that achieving 100% accuracy in spatially predicting future spermatophyte family diversity is challenging due to various factors. Nevertheless, the model predicts the general trend of increased spermatophyte family diversity under a single climate change scenario.

## 6 Conclusion

The SSMSFD model, based on the assumption that changes in climatic and geographic factors, as well as related processes such as species invasions and interactions, are the primary drivers of diversity patterns in nature reserves, allows us to separate the effects of global climate change on plant diversity from other factors. Among the 23 bioclimatic and geospatial variables considered, annual average precipitation has the most significant contribution to regional spermatophyte family diversity. Conversely, the monthly mean diurnal temperature difference has a negative effect on regional spermatophyte family diversity. The SSMSFD model explains 83.4% of the variation in regional spermatophyte family diversity using space as a substitute for time. Currently, the average diversity of spermatophyte families in China is approximately 118 families per 25 km^2^ of land, and this value tends to decrease from southeast to northwest. The effects of global climate change, excluding direct anthropogenic disturbances, have a positive impact on spermatophyte family diversity under the SSPs245 and SSPs585 scenarios. Previous studies have shown contrasting results regarding the impact of climate change on plant biomass and diversity, with some predicting an increase and others a decline [38,39]. These discrepancies can be attributed to the different levels of anthropogenic disturbance experienced by different ecosystems. It is evident that anthropogenic disturbances have a significant negative impact on plant diversity. The combined effect of global climate change and anthropogenic activities on plant diversity will result in a complex pattern of change. While the prediction has limitations, the SSMSFD model provides valuable insights into the quantitative assessment and prediction of seed plant family diversity.

## Data availability

Data are available on request to the authors.

## Ethics declarations

All participants/patients (or their proxies/legal guardians) provided informed consent to participate in thestudy. All participants/patients (or their proxies/legal guardians) provided informed consent for the publication oftheir anonymised case details and images.

## CRediT authorship contribution statement

**Yanzi Wang:** Writing – original draft, Validation, Software, Resources, Methodology, Investigation, Formal analysis, Data curation, Conceptualization. **Zhenhong Wang:** Writing – review & editing, Visualization, Supervision, Project administration, Funding acquisition.

## Declaration of competing interest

The authors declare that they have no known competing financial interests or personal relationships that could have appeared to influence the work reported in this paper.
